# Development of the assessment standards of the International Classification of Functioning, Disability, and Health (ICF) Geriatric Core Set through a modified Delphi method

**DOI:** 10.1186/s12877-024-04816-6

**Published:** 2024-03-07

**Authors:** Malan Zhang, Yan Gao, Jingjing Xue, Kun Li, Lifang Zhang, Jiani Yu, Tiebin Yan, Xiaohui Hou

**Affiliations:** 1 Department of Exercise Rehabilitation, College of Exercise and Health, Guangzhou Sport University, Guangzhou, China; 2 Department of Rehabilitation Medicine, Shenzhen Second People’s Hospital, First Affiliated Hospital of Shenzhen University, Shenzhen, China; 3 Department of Rehabilitation Medicine, Sun Yat-sen Memorial Hospital, Sun Yat-sen University, Guangzhou, China; 4 School of Nursing, Sun Yat-sen University, Guangzhou, China; 5 School of Nursing, Youjiang medical university for nationalities, Baise, China; 6 Department of Rehabilitation, GuangDong Province Hospital of Chinese Medicine, Guangzhou, China

**Keywords:** Delphi method, ICF, Geriatrics, Functional assessment

## Abstract

**Background:**

There is currently a lack of functional assessment tools based on the International Classification of Functioning, Disability, and Health (ICF) theoretical framework that are specific for older adults.

**Objective:**

The aim of the present study was to develop Chinese assessment standards of the ICF Geriatric Core Set for functional evaluation of older adults.

**Methods:**

A two-stage study process was conducted to develop the assessment standards of the ICF Geriatric Core Set: establishment of candidate assessment standards, and a modified Delphi consensus process including a pilot survey and two-round formal expert survey. Thirty participants in the field of ICF and geriatric rehabilitation were recruited. The suitability of the assessment standards in the questionnaires was rated using a Likert 5-level scoring method. The arithmetic mean, the full mark ratio and the coefficient of variation (CV) were used as screening indicators for the assessment standards, and modification was made for several standards, in line with the Delphi results and the expert panel discussion.

**Results:**

Thirty-three candidate assessment standards belonging to 17 categories were generated. A total of 26 and 24 experts in the field of ICF and geriatric rehabilitation participated in the two-round survey, respectively. Five standards belonging to four categories entered into the second-round survey directly, five standards belonged to five categories entered with minor modification, and nine standards belonging to seven categories were redesigned based on the literature and discussion of the expert panel. In the second-round survey,15 assessment standards belonging to 15 categories met the screening requirements and four assessment standards belonged to the two remaining categories that needed a criterion and which the expert panel discussed for the final decision.

**Conclusions:**

Using the modified Delphi method, the assessment standards of the ICF Geriatric Core Set have been developed.Future work should focus on the reliability and validity of the the assessment standards and their application to the health management of older adults.

**Supplementary Information:**

The online version contains supplementary material available at 10.1186/s12877-024-04816-6.

## Background

Since the beginning of the 21st century, the process of global population aging has accelerated significantly. The proportion of older people in the global population is expected to approach or exceed 22% by 2050 [1]. China has entered the rapid development stage of population aging. It is estimated that the proportion of older adults in China will exceed 20% in 2024, reach 30% in 2039, and increase to 34.9% in 2053, when the number of older adults will reach a peak of 487 million [2]. In addition, the number of older adults with disability reached 52.7 million in 2020, and it has been estimated that the proportion of disabled older adults in China will account for over 57% of total disabled persons by 2030 and over 70% by 2050 if no prevention and control measures are implemented [3]. The increasing number of older individuals with disability has led to a dramatic increase in the demand for rehabilitation and long-term care services, placing a heavy burden on families, societies, and even the country [4]. For promoting the development of healthy aging, it is essential to accurately grasp the functional characteristics of older adults, carry out personalized rehabilitation, and meet the needs of multidimensional rehabilitation and care services such as physical and psychological care for older adults [5]. Although there are some functional assessment tools developed for older adults, most of them are applicable to certain diseases or only cover physical function and activity [6–9]. There is scarce literature on composite and universal functional assessment tools that take into account multiple dimensions of body structure, function, activity, participation, and environmental factors in older adults specifically.

The International Classification of Functioning, Disability, and Health (ICF) is a theoretical framework and a classification system for describing health and health status that was promulgated by the World Health Organization (WHO) in May 2001 [10]. The ICF is an internationally unified tool for functional assessment and description [11, 12]. It is based on a “biological-psycho-social medical model,” which unifies health and disability as a multidimensional integrated whole of human functioning, with the core concept being that an individual’s functioning in a given domain depends on the interaction between health conditions and contextual (environmental and personal) factors. Compared with other assessment tools, the ICF adds dimensions such as body structure and environmental factors, which can more comprehensively evaluate the functional level of service objects. To further promote the application of the ICF in practice, the WHO has introduced a series of ICF Core Sets [13, 14]. Among them, the ICF Rehabilitation Set (ICF-RS) belongs to the general ICF Core Set for functional evaluation [15]. As the entire ICF Core Set introduced by the WHO is only a list of categories and lacks specific operational standards, our ICF research team has developed the assessment standard of the ICF-RS, which has been clinically verified to have good reliability and validity [16–18]. The assessment standard of the ICF-RS has been published as a national standard (GB/T41843-2022), which has been recommended to be used as a universal function assessment tool for the rehabilitation of adult population in China. The ICF Geriatric Core Set (ICF-GS) is a functional evaluation tool endorsed by WHO specifically for older adults [19]. It consists of 38 categories and lacks the corresponding assessment standards. Although the ICF-RS and ICF-GS overlap in 14 categories and can directly use the corresponding Chinese standards, the remaining categories of ICF-GS still lack criteria for functional assessment, which leads to difficulties in practical application. The Delphi method is associated with obtaining a group decision from a group of experts [20], and the modified Delphi technique consisting of a self-administered questionnaire and the discussion of the results has been widely used to develop the new Core sets and assessment standards related to **ICF** [17, 21]. The purpose of the study was to further develop the assessment standards of the ICF-GS (simplified version), so as to provide an auxiliary tool based on the ICF framework for the functional evaluation of older adults.

## Methods

### Study design

We used a modified Delphi approach to obtain a consensus opinion on the Chinese assessment standards of the ICF-GS. The study process consisted of two stages, namely establishment of candidate assessment standards and a modified Delphi consensus process.

### Categories of the ICF-GS (simplified version)

The evaluation criteria of the ICF-GS were further developed based on the Chinese assessment standards of the ICF-RS. The simplified version of the ICF-GS contains 38 categories, including seven categories for body structure, seven categories for body function, 15 categories for activities and participation, and nine categories for environmental factors [19] (Table 1). The ICF-GS and ICF-RS overlap in 14 categories (Appendix 1). For the overlapping categories of the ICF-GS, the existing assessment standards can be used directly. In addition, body structure can be directly assessed based on the nature, extent, and location of injury. However, there are 17 other categories, including four items of physical function, four items of activities and participation, and nine items of environmental factors (Table 1), that need to be supplemented with assessment standards.

**Table 1:** The list of categories of the ICF-GS

Body structure (n = 7)	Body function (n = 7)	Activities and participation (n = 15)	Environmental factors (n = 9)
s110 Structure of brain	* b630 Sensations associated with urinary functions	* d760 Family relationships	*e110 Products or substances for personal consumption
s320 Structure of mouth	* b460 Sensations associated with cardiovascular and respiratory functions	* d860 Basic economic transactions	*e245 Time-related changes
s430 Structure of respiratory system	* b435 Immunological system functions	* d460 Moving around in different locations	*e330 People in positions of authority
s610 Structure of urinary system	* b765 Involuntary movement functions	* d360 Using communication devices and techniques	*e355 Health professionals
s720 Structure of shoulder region	^#^b130 Sleep functions	^#^d230 Carrying out daily routine	*e425 Attitudes of acquaintances, peers, colleagues, neighbors, and community members
s750 Structure of lower extremity	^#^b455 Exercise tolerance functions	^#^d410 Changing basic body position	*e450 Individual attitudes of acquaintances, peers, colleagues, neighbors, and community members
s770 Additional musculoskeletal structures related to movement	^#^b620 Urination functions	^#^d415 Maintaining a body position	*e460 Societal attitudes
		^#^d420 Transferring oneself	*e465 Social norms, practices, and ideologies
		^#^d450 Walking	*e570 Social security services, systems, and policies
		^#^d465 Moving around using equipment	
		^#^d510 Washing oneself	
		^#^d520 Caring for body parts	
		^#^d530 Toileting	
		^#^d550 Eating	
		^#^d570 Looking after one’s health	

*Refers to the categories that require additional assessment standards

^#^Refers to the categories that overlap with ICF-RS

### Stage 1: establishment of candidate assessment standards for the ICF-GS

The following two methods were used to establish candidate assessment standards for the ICF-GS [17]: ① Foreign databases (PubMed, Embase, and Web of Science) and Chinese databases (CNKI, Wanfang, and China Biomedical Literature Database) were searched. The items of the assessment scales reported in the literature were associated with the categories of the ICF-GS using the ICF linking rules [22], and the former assessment content was used as a candidate assessment standard after the linking. ② We developed part of the assessment standards in line with the definition of ICF-GS. The characteristics and needs of the older adults were fully considered in the process of developing the assessment standards. A full-time ICF researcher preliminarily developed assessment standards for each category and screened them one by one. The screening principles included the representativeness and the suitability of the evaluation methods. The candidate assessment standards for each category to be included in the expert survey were determined after screening by a professor and chief physician in the field of rehabilitation medicine and ICF. In order to control the expert survey time, no more than three assessment standards were eventually included for each category.

### Stage 2: Delphi consensus process design of the expert survey questionnaire

The expert survey questionnaire consisted of three parts as follows: ① invitation letter, containing the detailed description of the background, purpose, and significance of the research and the invitation made for the survey; ② general information questionnaire for experts, including the expert’s personal basic information (name, age, gender, education, profession, title, years engaged in rehabilitation, years using the ICF), the expert’s familiarity with ICF, and the basis for judgment; ③ expert consultation questionnaire, including the code, definition, description, candidate assessment standards of the ICF-GS categories, and notes for filling. Each category had two or three candidate assessment standards, and each standard listed the corresponding evaluation questions, rating criteria, judgment column using a Likert 5-level scoring method [23], and expert opinion column.

### The pilot survey

Before the formal survey began, five experts were invited to participate in the preliminary survey. The pilot survey included one senior professor and chief physician in the field of the ICF, three associate chief nurses, and one intermediate therapist, who have used the ICF in research or work for more than five years, mainly serving older adults. According to the opinions and suggestions of these experts, the researchers modified the questionnaire and formed the official version of the expert consultation questionnaire.

### Participants

**Fig. 1:**
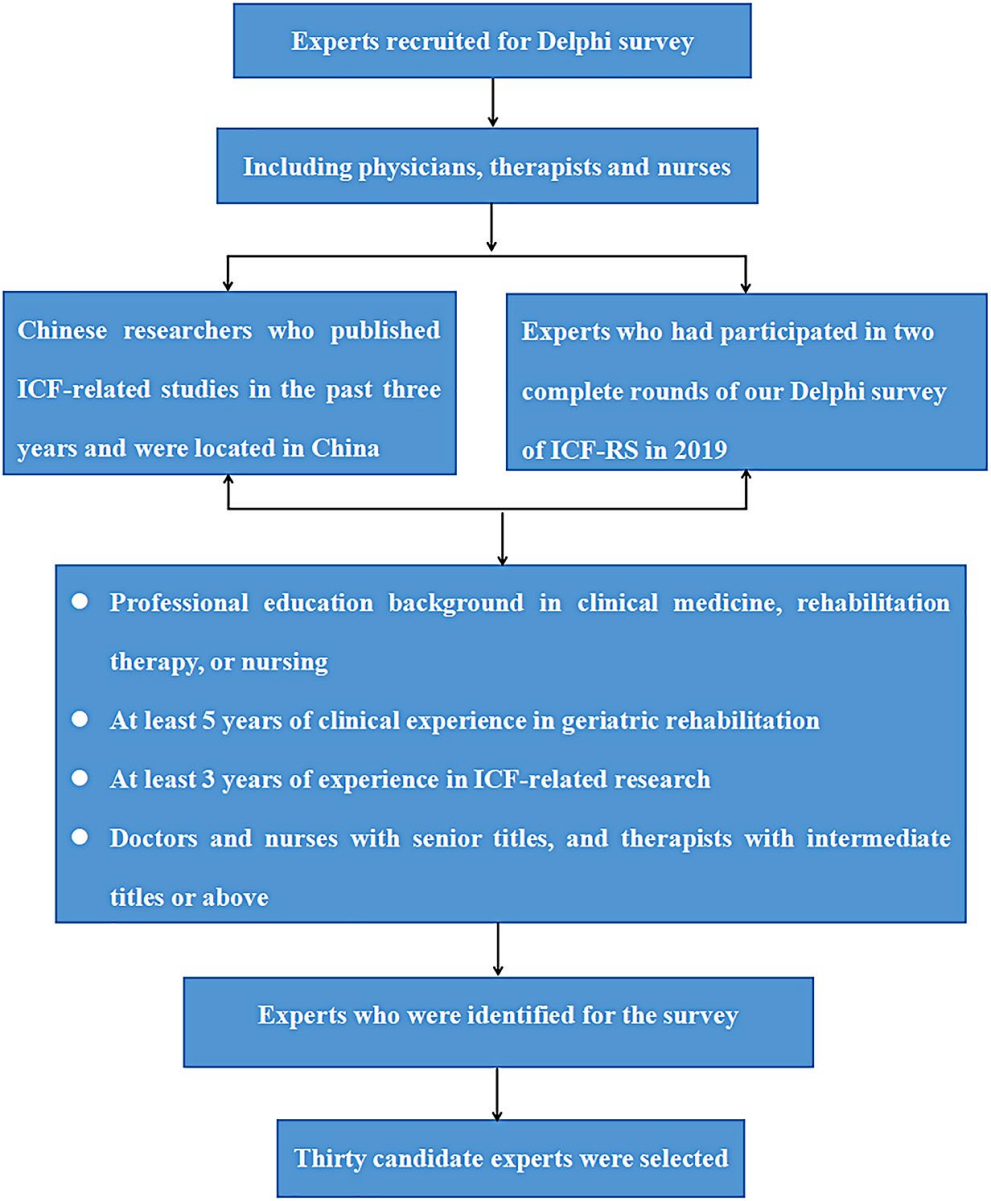
The procedure of selecting experts for the Delphi survey

The sampling method of the expert survey included purposive sampling and snowball sampling [24]. Specifically, the list of experts who participated in the survey was compiled in the following three ways: ① Chinese researchers, including physicians, nurses, and therapists, who were located in China and published ICF-related studies in foreign databases (such as PubMed, Embase, and Web of Science) and Chinese databases (such as CNKI and Wanfang database) were searched; ② The experts who had participated in [ 25] with the research direction of geriatric rehabilitation medicine were included; ③ Experts who were identified for the survey recommended others. The identified experts had to meet the following conditions: ① professional education background in clinical medicine, rehabilitation therapy, or nursing; ② at least 5 years of clinical experience in geriatric rehabilitation; ③ at least 3 years of experience in ICF-related research; ④ doctors and nurses with senior titles, and therapists with intermediate titles or above. According to previous literature, the number of experts should be 15–30 [25]. This study intended to include 30 ICF experts, and geographical representation was taken into account in the recruitment process. Figure 1 shows the procedure for selecting experts.

### The first round of expert survey

The first round of expert survey included three steps, namely expert invitation, questionnaire filling, and questionnaire collection. The ICF researcher who had developed the assessment standards first used mobile communication applications (WeChat) to contact each expert with the invitation letter and asked if they would participate in the survey, and then the general information questionnaire and expert consultation questionnaire were sent to the experts who confirmed their participation in the survey. The experts had to assess the suitability of each candidate standard on a 5-point scale from“strongly unsuitable”(quantified as 1) to“strongly suitable”(quantified as 5). If the experts had some advice for the evaluation question, rating criteria of each standard, they could fill in the expert opinion column. Only one result was allowed to be selected for each candidate standard. Multiple selections and missing items for each candidate standard were considered invalid. It took approximately 20 to 30 min to complete the questionnaire.

The experts were asked to fill in the questionnaire within two weeks. Three days before the survey deadline, the researchers reminded the experts who had not yet completed the questionnaire through WeChat. All questionnaires were collected and checked by the researchers. If there were any omissions, the experts were contacted to resolve them.

### The second round of expert survey

The expert questionnaire of the second-round survey was generated in line with the statistical results and experts’ modified opinions. The experts who participated in the first-round survey received a second questionnaire by WeChat. The questionnaire reported the first round scores and expert opinions for each category. The changes in each assessment standard were highlighted in red. The experts were asked to consider the suitability of each assessment standard using a 5-point Likert scale from“strongly unsuitable” to “strongly suitable”. The experts could also fill in the comments column for modification. They were given two weeks to return the responses, and a reminder was sent three days before the deadline by the researchers.

### Data analysis

#### The positive coefficient and the authority coefficient

Excel was used to manage the data, and SPSS 22.0 software was used for statistical processing. Frequency (percentage) or mean (standard deviation) were used to describe the basic information of the experts and the suitability value of each candidate standard. We calculated the positive coefficient and the authority coefficient of the experts. As for the experts’ positive coefficient, the recovery rate of the expert survey can reflect the degree of experts’ concern and cooperation for the research [26]. A recovery rate of 50% allows to analyze the data, and a recovery rate over 70% is very good [27]. The experts’ authority coefficient (Cr) is the arithmetic mean of the experts’ judgement coefficient (Ca) and the experts’ familiarity degree (Cs), namely Cr=(Ca + Cs)/2. Generally, an authority coefficient of ≥ 0.7 is acceptable [26]. Experts’ judgment coefficient (Ca) can be quantified from four aspects, namely practical experience, theoretical analysis, reference to domestic and foreign data, and intuition [26, 28]. The evaluation criteria are shown in Appendix 2 [24]. Familiarity (Cs) was self-rated by the experts on their familiarity with the survey content [28]. The average degree of familiarity of the consulting experts was calculated based on a Likert scale method to rate the familiarity with the question on a scale from 0 to 1 (1 = very familiar, 0.8 = more familiar, 0.6 = generally familiar, 0.4 = not very familiar, 0.2 = not familiar) [24].

### The concentration degree and the coordination degree

The screening indicators of the assessment standards for each ICF category included the concentration degree and the coordination degree of expert opinions [29]. The concentration degree of expert opinions was expressed by the arithmetic mean and the full mark ratio of the suitability score of each category. The full mark ratio of the suitability score was the proportion of the total number of experts who considered the standards to be somewhat suitable, fairly suitable, or very suitable. The coordination degree of expert opinion was expressed by the coefficient of variation (CV), which described the variation degree of the evaluation results in a single index; it was calculated as the standard deviation divided by the mean. Smaller values indicated a higher degree of coordination among the expert opinions [30]. The detailed screening criteria were as follows [31, 32]: ① After the first-round survey, the standards were removed if the full mark ratio was lower than 20%; ② For one category, if the mean value of suitability degree in the candidate standard was ≥ 4 and the coefficient of variation was < 25%, the candidate standard was directly entered into the second round of the expert survey, while the other candidate standards were eliminated; ③ If the mean value of the suitability degree was ≥ 3.5 and < 4, while the coefficient of variation was < 25%, the proposed candidate standard for this category was revised by the panel of five experts with reference to the opinions of other experts in the first-round survey; ④ If none of the candidate standards for one category met the conditions that the mean value of suitability degree was ≥ 3.5 and the coefficient of variation was < 25%, then the candidate standards for that category were redesigned according to the literature and the experts’ opinions.

The candidate criteria for final retention were the mean value of suitability degree of the candidate standard > 3.5 and the coefficient of variation < 25%. If less than 10% of the categories remained without established standards, the next round of the expert survey was not conducted. If a category had more than one standard that met the criteria, the final standard was determined through discussion of the expert panel including the five experts who had participated in the pilot survey.

## Results

### Preliminary formulation of assessment standards for the ICF-GS

Finally, a total of 33 candidate assessment standards entered the first round of the expert survey. Specifically, two categories included three candidate standards, six categories included two candidate standards, and nine categories included one candidate standard.

### Results of the Delphi survey

#### The pilot survey

In June 2022, an expert panel including five experts participated in the pilot survey. All of the five experts used the ICF in research or work for more than 5 years. In the pilot survey, the experts put forward their own suggestions on the Chinese translations of the ICF categories in the questionnaire, the evaluation questions, and the rating criteria of the generated standards, and composed the final expert consultation questionnaire.

### Basic information of the experts

Of the 30 experts selected, three did not respond to the invitation, and one of the experts accepted the invitation but did not finish the questionnaire due to a busy schedule. Finally, a total of 26 experts from 11 provinces of Guangdong, Jiangsu, Hubei, Jiangxi, Shaanxi, Anhui, Shanxi, Fujian, Hunan, Sichuan, Guangxi, and two municipalities directly under the central government of Shanghai and Beijing participated in the first round of the expert survey. The response rate of the survey was 96.3%, the efficiency of the questionnaire was 100%, and the experts’ positive coefficient was 96.3%. In the second round, a total of 26 questionnaires were sent and 24 questionnaires were recovered, that is, the recovery rate of expert consultation forms was 92.3%, and the experts’ positive coefficient was 92.3%. Among the 26 experts involved in this study, there were 10 doctors, 6 nurses, and 10 therapists. The experts were 34–66 years old (mean age, 42.6 ± 7.1 years). They had been engaged in older adults rehabilitation for 5–31 years (mean 15.2 ± 5.5 years) and exposed to ICF for 3–15 years (mean 8.0 ± 4.0 years). Twenty individuals had a master’s degree or above (76.9%), and 17 of them had a title of vice-senior or above (65.4%). The detailed information of the experts is shown in Table 2. The experts’ authority coefficient was 0.750–1.000 (median 0.900, interquartile 0.887–0.900) (Table 2).

**Table 2:** Demographic characteristics and professional experience of experts (*N* = 26)

Items	Frequency	Percentage (%)	Mean ± SD
**Sex**			
Men	14	53.8%	
Women	12	46.2%	
Age (years)			42.6 ± 7.1
≤ 39	11	42.3%	
40–60	15	55.7%	
**Profession**			
Physician	10	38.5%	
Nurse	6	23.1%	
Therapist	10	38.4%	
**Professional title**			
Attending	9	34.6%	
Vice-senior	9	34.6%	
Senior	8	30.8%	
**Education background**			
Bachelor	6	23.12%	
Master	7	26.9%	
Doctor	13	50%	
**Working experience in rehabilitation (years)**			15.2 ± 5.5
< 10	3	11.5%	
10–19	17	65.4%	
20–31	6	23.1%	
**ICF experience (years)**			8.0 ± 4.0
3–5	10	38.5%	
6–15	16	61.5%	
**Frequency of applying ICF at work**			
Occasionally	12	46.2%	
Frequently	14	53.8%	
**Expert authority coefficient**			
0.90–1.00	20	76.9%	
0.80–0.89	4	15.4%	
0.70–0.79	2	7.7%	

### Results of the first and second-round survey

The full mark ratio of the candidate standards was 69.2–96.2% in the first round and 87.5–100% in the second round of the expert survey. In the first round of the expert survey, there were four categories with five candidate assessment standards that met the criteria of the mean value of the suitability degree > 4 and the coefficient of variation < 25%. The corresponding candidate assessment standards of those categories were directly included in the second round of the expert survey. Among them, the category “b630 Sensations associated with cardiovascular and respiratory functions” had two candidate standards. The categories “b765 Involuntary movement function”, “d860 Basic economic transactions” and three environmental factors each had one candidate assessment standard that met the conditions of mean value of the suitability degree between 3.5 and 4 and the coefficient of variation < 25%, and these standards were entered to the second round of the expert survey with minor modification, which was discussed by the panel of five experts with reference to the other experts’ opinions in the first-round survey. A total of nine candidate standards of “b630 Sensations associated with urinary functions”, “b435 Immunological system functions” and five environmental factors did not meet the conditions that the mean value of the suitability degree was greater than 3.5 and the coefficient of variation was less than 25%. Those assessment standards were redesigned based on the literature and discussion of the expert panel. According to the feedback of most experts, the assessment terms of the five environmental factors were too raw, abstract and not easily understandable for the older adults. Modification was made using more accessible and colloquial words without changing the original content.

**Fig. 2:**
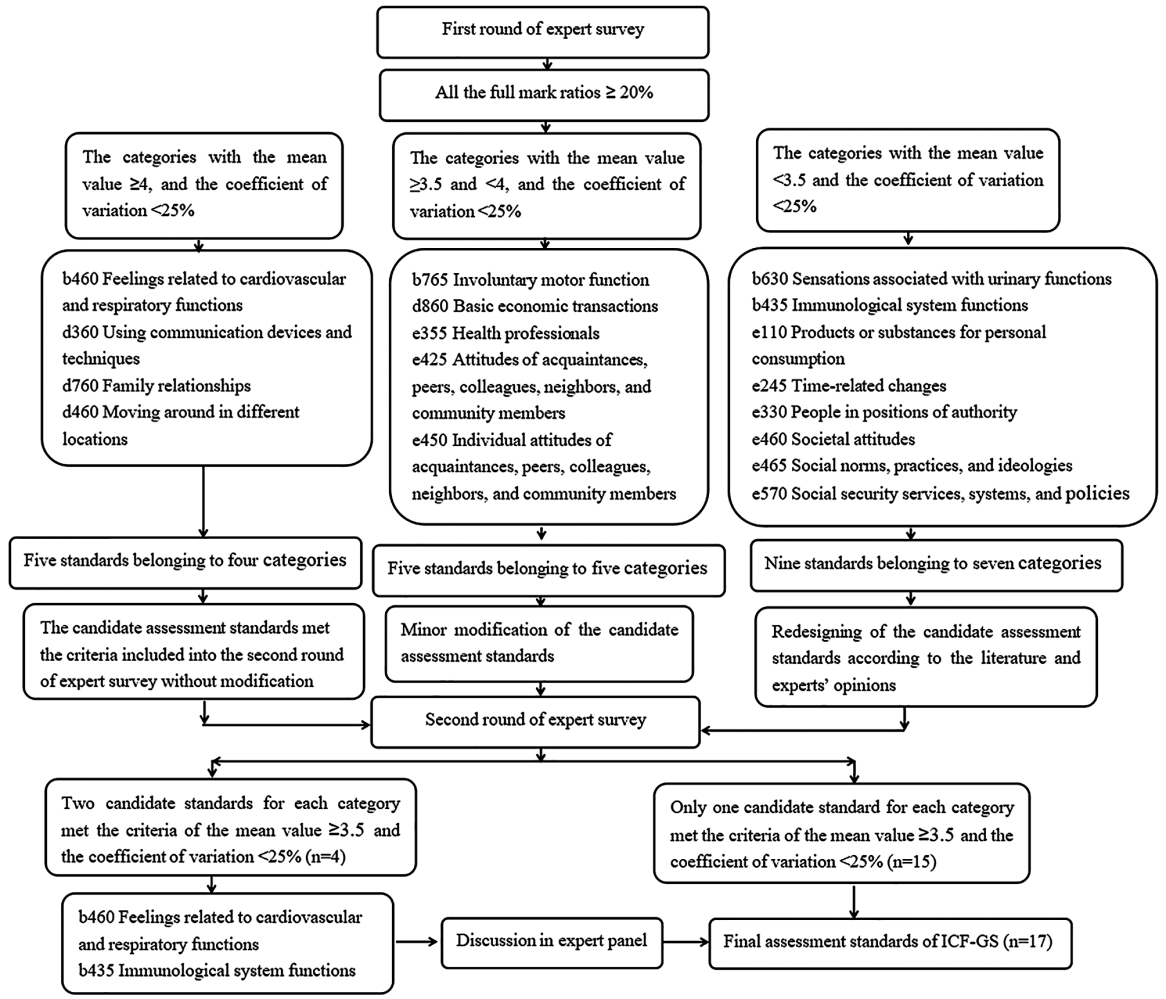
The procedure of the two rounds of the expert survey

In the second round of the expert survey, all of the candidate standards were in conformity with the mean value of the suitability degree > 3.5 and the coefficient of variation < 25%, with the mean range of 3.88–4.33 (total score: 5). After the second round of the expert survey, there were still two candidate criteria included in the categories “b630 Sensations associated with cardiovascular and respiratory functions” and “b435 Immunological system functions”, and their final content was determined through discussion at the expert panel. The procedure of the two rounds of the expert survey is shown in Fig. 2. Specific statistical results are shown in Table 3.

**Table 3:** Scoring results of the two rounds of the expert survey

ICF categories		The first round				The second round			
		Mean value	Standard deviation	Full mark ratio	Coefficient of variation	Mean value	Standard deviation	Full mark ratio	Coefficient of variation
**b460** Sensations associated with cardiovascular and respiratory functions	Candidate 1	3.46	0.811	88.4%	23.4%				
	Candidate 2	4.15	0.881	92.3%	21.2%	4.21	0.721	100%	17.1%
	Candidate 3	4.15	1.008	92.3%	24.3%	4.29	0.806	95.8%	18.8%
**b630** Sensations associated with urinary functions	Candidate 1	3.81	1.167	80.8%	30.6%	4.13			
	Candidate 2	3.69	1.087	84.6%	29.5%	3.58	0.776	100%	21.7%
**b765** Involuntary movement functions	Candidate 1	3.81	0.939	92.3%	24.6%	4.13	0.612	91.7%	14.8%
	Candidate 2	3.96	0.916	92.3%	23.1%	3.46	0.884	100%	25.5%
**b435** Immunological system functions	Candidate 1	3.58	0.902	92.3%	25.2%	3.92	0.929	87.5%	23.7%
	Candidate 2	3.27	1.151	73.1%	35.2%	3.92	0.83	91.7%	21.2%
**d360** Using communication devices and techniques	Candidate 1	4.04	0.99	92.3%	24.7%	4.0	0.722	95.8%	18%
	Candidate 2	3.5	1.03	76.9%	29.4%				
**d760** Family relationships	Candidate 1	3.46	0.989	80.8%	28.6%				
	Candidate 2	4.12	0.952	92.3%	23.1%	4.21	0.658	95.8%	15.7%
**d860** Basic economic transactions	Candidate 1	3.85	0.925	96.2%	24.0%				
	Candidate 2	3.62	0.852	92.3%	23.5%				
	Candidate 3	4.04	0.999	88.4%	24.7%	4.33	0.637	100%	14.7%
**d460** Moving around in different locations	Candidate 1	3.85	0.925	96.2%	24%	4.29	0.806	100%	18.8%
	Candidate 2	4.04	0.958	92.3%	23.7%				
**e110** Products or substances for personal consumption	Candidate 1	3.42	1.137	69.2%	33.2%	3.92	0.83	95.8%	21.2%
**e245** Time-related changes	Candidate 1	3.27	0.962	69.2%	29.4%	3.75	0.897	91.7%	23.9%
**e330** People in positions of authority	Candidate 1	3.50	1.030	80.8%	29.4%	3.96	0.751	100%	19.0%
**e355** Health professionals	Candidate 1	3.96	0.916	88.4%	23.1%	4.17	0.761	100%	18.2%
**e425** Attitudes of acquaintances, peers, colleagues, neighbors, and community members	Candidate 1	3.96	0.958	88.4%	24.2%	4.21	0.721	100%	17.1%
**e450** Individual attitudes of acquaintances, peers, colleagues, neighbors, and community members	Candidate 1	3.73	0.874	84.6%	23.4%	4.04	0.751	100%	18.6%
**e460** Societal attitudes	Candidate 1	3.38	1.098	69.2%	32.5%	3.88	0.900	87.5%	23.2%
**e465** Social norms, practices, and ideologies	Candidate 1	3.23	0.992	73.1%	30.7%	3.96	0.69	100%	17.4%
**e570** Social security services, systems, and policies	Candidate 1	3.85	1.008	84.6%	26.2%	4.08	0.83	100%	20.3%

## Discussion

China is facing an increasingly severe aging trend. The proportion of the older adults and disabled older adults has increased rapidly, and the disbalance between the supply and demand of medical care and old-age care has become increasingly prominent [33]. Older adults often have many chronic diseases, various complications, complex conditions, and other characteristics. Accurate assessment and intervention services for health management of the older adults can maximize the recovery of the declining physical function, to meet the needs of disabled older adults for diversified rehabilitation and old-age care. Therefore, to carry out health management for the older adults, it is first necessary to establish functional assessment tools and standardized assessment criteria of functional impairment for the older adults [34]. The WHO recommends the use of the ICF Core Set to analyze dysfunction and rehabilitation needs and guide the development of rehabilitation programs [13]. Rehabilitation intervention and rehabilitation cycle management are carried out on the basis of the ICF functional evaluation, combined with the International Classification of Health Intervention (ICHI) [35, 36]. This study finally developed Chinese assessment standards of the ICF-GS for the older adults.

The Chinese assessment standards of the ICF-GS could be used for the functional assessment and health management of the older adults, including body structure, physical function, activity & participation, and environmental factors [19]. Compared with other assessment tools such as the modified Barthel scale [37, 38], the theoretical framework of the ICF covers body structure and environmental factors, and has more multidimensional, dynamic, and bidirectional characteristics, so it can be used as an effective supplement for the functional assessment of the older adults.

In this paper, two rounds of the modified Delphi method were used to develop the assessment standards of the ICF-GS. The developed assessment standards met the criteria that the mean value of the suitability degree was > 3.5 and the coefficient of variation was < 25%, which have been reported in previous literature [39, 40]. The number of experts participating in the survey met the required number. In two rounds of expert surveys, the positive coefficient was 96.3% and 92.3%, and the authority coefficient was between 0.750 and 1.000, which met the standard of ≥ 0.7 proposed by Collier et al. [41], indicating that the results of the expert survey have good authority and reliability. After the second round of the expert survey, two candidate standards were still included in the category “b460 Sensations associated with cardiovascular and respiratory functions.” The standard corresponding to the New York Heart Association (NYHA) scale was selected out by the expert panel as it paid attention to both the sensations of the heart and breath, compared with the other standard corresponding to the modified Medical Research Council (mMRC) dyspnea scale [42, 43]. “b435 Immunological system functions” also included two candidate standards, and the final standard was chosen by the experts on the basis of what would be easier for older people to understand.

In this study, the modified Delphi method was used to develop the assessment standards of the ICF-GS. The Delphi method is a quantitative and qualitative forecasting and evaluation method that extensively solicits the opinions of experts in an anonymous way, and gradually consolidates the opinions of experts through repeated information exchange and feedback correction [44, 45]. The classical Delphi method, especially in the first round of expert survey, mostly adopts the form of an open questionnaire with discrete opinions, which is difficult for statistical analysis and easily introduces subjectiveness to statistical analysts. The modified Delphi method can directly adopt the form of a scale, and the designed content is broad but moderately limited. In addition, compared with the classical method, the modified Delphi method reduces the number of cycles. The consultation can be ended as soon as the opinions of experts have become consistent, rather than insisting on four rounds [46]. At present, there are many studies with the modified Delphi survey, and they are mainly related to the development of standards, evaluation schemes, and scales [47–48]. In this study, two rounds of the modified Delphi expert survey and expert discussion were used to form the final version of the assessment standards of the ICF-GS.

There are some limitations to this study. First, due to time constraints, qualitative analysis such as semi-structured interview was not conducted on the developed assessment standards of the ICF-GS. Second, related studies have not been conducted on the availability, reliability, and validity of the developed assessment standards in the clinical use; therefore, comparisons with the present study were not possible.

## Conclusion

This study discussed the development process of the quantitative standard of the ICF-GS (simplified version), and provided an auxiliary tool based on the ICF theoretical framework for the functional assessment of the older adults. Since the ICF-GS covers four dimensions such as environmental factors, it can assess the functional status of older adults population from a more comprehensive perspective, and can be a favorable supplement to other functional assessment tools. The Chinese assessment standard of ICF-GS may help to investigate the functional characteristics of older adults, formulate health strategies, and evaluate the rehabilitation effect in China.

### Electronic supplementary material

Below is the link to the electronic supplementary material.

Supplementary Material 1. List of the ICF-RS with 30 categories

Supplementary Material 2. The evaluation cretieria of the Experts’ judgment coefficient (Ca)

## Data Availability

The data generated and analyzed are not publicly available to preserve the anonymity of the participants, but they are available from the corresponding author (Xiaohui Hou) on a reasonable request.

## Abbreviations

ICF: International Classification of Functioning, Disability, and Health.
ICF-RS: International Classification of Functioning, Disability, and Health Rehabilitation Set.
ICF-GS: International Classification of Health Intervention.
Cr: The experts’ authority coefficient.
Ca: The experts’ judgement coefficient.
Cs: The experts’ familiarity degree.
