# Supplementary material for: Development of the assessment standards of the International Classification of Functioning, Disability, and Health (ICF) Geriatric Core Set through a modified Delphi method
Source: BMC Geriatr. 2024 Mar 7;24:239. doi: 10.1186/s12877-024-04816-6 (PMC10921752; doi:10.1186/s12877-024-04816-6)
Supplement: Supplementary file 1 — Supplementary Material 1. List of the ICF-RS with 30 categories [file 12877_2024_4816_MOESM1_ESM.doc]

**Appendix 1**

**Table 1**  List of the ICF Rehabilitation Set with 30 categories

| **Body function(n=9)** | **Activity(n=14)** | **Participation(n=7)** |
| --- | --- | --- |
| b130 Energy and drive functions | d240 Handling stress and other psychological demands | ^#^d230 Carrying out daily routine |
| ^#^b134 Sleep functions   |  | | --- | | ^#^d410 Changing basic body position | d470 Using transportation |
| b152 Emotional functions | ^#^d415 Changing basic body position | d660 Assisting others |
| b280 Sensation of pain   |  | | --- | | ^#^d420 Transferring oneself | d710 Basic interpersonal interactions |
| ^#^b455 Exercise tolerance   | functions | | --- | | ^#^d450 Walking | d770 Intimate relationships |
| ^#^b620 Urination functions | ^#^d465Moving around using equipment | d850 Remunerative employment |
| b640 Sexual functions | d455 Moving around | d920 Recreation and leisure |
| b710 Mobility of joint functions | ^#^d510 Washing oneself |  |
| b730 Muscle power functions | ^#^d520 Caring for body parts |  |
|  | ^#^d530 Toileting |  |
|  | d540 Dressing |  |
|  | ^#^d550 Eating |  |
|  | ^#^d570 Looking after one’s health |  |
|  | d640 Doing housework |  |

**^#^Refers to the categories that overlap with ICF-GS**
