# Supplementary material for: Development of the assessment standards of the International Classification of Functioning, Disability, and Health (ICF) Geriatric Core Set through a modified Delphi method
Source: BMC Geriatr. 2024 Mar 7;24:239. doi: 10.1186/s12877-024-04816-6 (PMC10921752; doi:10.1186/s12877-024-04816-6)
Supplement: Supplementary file 2 — Supplementary Material 2. The evaluation cretieria of the Experts’ judgment coefficient (Ca) [file 12877_2024_4816_MOESM2_ESM.doc]

**Appendix 2**

To assist us in the reliability analysis of expert consultation. Please help us to fill in the following:

How long have you been in contact with ICF? ______ years

1. Have you ever used ICF in your work or research?

□ Never used □ Occasionally used □ Often used

2. Please rate your familiarity with the ICF core categories :(Please mark "√" next to the corresponding description)

□ No □ Not familiar □ General □ Familiar □ Familiar

3. Please make a judgment and select the degree of influence according to the following judgment criteria (Please mark "√" with the corresponding description)

**Table 1** Judgement basis and the degree of influence

| **Judgement basis** | **The degree of influence** | | | |
| --- | --- | --- | --- | --- |
|  | **Large** | **Medium** | | **Small** |
| Practical experience |  | |  |  |
| Theoretical analysis |  | |  |  |
| Reference to domestic and foreign data |  | |  |  |
| Intuition |  | |  |  |

**Different levels of influence are scored as follows**

**Table 2** Judgement basis and the degree of influence

| **Judgement basis** | **The degree of influence** | | | |
| --- | --- | --- | --- | --- |
|  | **Large** | **Medium** | | **Small** |
| Practical experience | 0.5 | | 0.4 | 0.3 |
| Theoretical analysis | 0.3 | | 0.2 | 0.1 |
| Reference to domestic and foreign data | 0.1 | | 0.1 | 0.1 |
| Intuition | 0.1 | | 0.1 | 0.1 |
